# Influence of Oxygen Management during the Post-Fermentation Stage on Acetaldehyde, Color, and Phenolics of *Vitis vinifera* L. Cv. Cabernet Sauvignon Wine

**DOI:** 10.3390/molecules27196692

**Published:** 2022-10-08

**Authors:** Lingmin Dai, Yuhang Sun, Muqing Liu, Xiaoqian Cui, Jiaqi Wang, Jiming Li, Guomin Han

**Affiliations:** 1 School of Bioengineering, Qilu University of Technology (Shandong Academy of Sciences), Jinan 250353, China; 2 Yantai Changyu Group Corporation Ltd., Yantai 264001, China

**Keywords:** wine, post-fermentation stages, oxidation, acetaldehyde, color, phenolics

## Abstract

Oxygen exposure is unavoidable and the impact of its management during the post-fermentation stage (PFS) on dry red wine is poorly investigated. This study was dedicated to the variation of acetaldehyde, color and phenolics of Cabernet Sauvignon dry red wine during five discontinuous oxidation cycles of four levels of controlled oxygen supply, which were carried out to simulate probable oxidation during the PFS. Free SO_2_ disappeared after the first, second and third oxidation cycles in wines with high, medium and low levels of oxygen exposure severally, but subsequent oxygen exposure below or equal to 2 mg O_2_/L per cycle had little effect while 3–3.9 mg O_2_/L per cycle dramatically facilitated acetaldehyde accumulation, which was accompanied by an enormous variation in color and pigments, especially when total oxygen consumption was above 10 mg/L. The utilization of clustered heatmap and partial least square regression demonstrated the feasibility of characterization of wine oxidation degree using the chemical parameters measured by UV-spectrophotometry. Oxygen exposure during the PFS should be emphatically controlled, and chemical indexes determined by the UV–spectrophotometric method can be used for a scientific and effective description of wine oxidation degree.

## 1. Introduction

The classic steps in red winemaking can be divided into four main steps: mechanical harvest treatments, maceration and alcoholic fermentation, separation of wine and pomace, and malolactic fermentation (MLF) [1]. However, the following PFSs such as clarification, racking, cold stabilization, coarse filtration and sterile filtration are also absolutely necessary. As the atmosphere consists of approximately 21 percent oxygen, oxygen exposure can occur at each step during the winemaking. During alcoholic fermentation, molecular oxygen is an essential nutritional factor that yeast cells need to produce sterols and unsaturated fatty acids, which significantly influences ethanol tolerance, fermentative capability and viability of yeast. By the end of alcoholic fermentation, lees containing considerable dead yeast and a small amount of survival yeast are formed; previous studies had shown that dead yeast had the ability to consume oxygen and the remaining living yeast also metabolized oxygen [2,3,4], hence higher levels of oxygen are not so terrible to wine chemical components just after alcoholic fermentation.

In the absence of information about oxygen consumption of red wines during the PFS, oxygen rates and doses taken from published trials about micro-oxygenation (mox) prior to (pre-) and after MLF best serve as guides to understanding the level of oxygen exposure during the wine PFS. Oxygen application rates and doses during wine mox treatments at these two different wine production stages were concerned on the basis of data obtained from the literature from 2001 to 2009, and the results showed that the mox rate and total oxygen additions were both much higher when mox is applied pre- rather than post-MLF [5]. This means that the ability of oxygen uptake is much lower in the wines after MLF, which could be attributed to lots of lees contained in the wine just after the end of alcohol fermentation, consequently reducing the oxidation of wine chemicals [3]. Generally, parts of the lees will be removed during inversion before and after MLF, which will significantly reduce the ability of a wine to consume oxygen. Furthermore, any excess acetaldehyde produced during pre-MLF mox can be eliminated by malolactic bacteria during the following MLF, but this useful side-effect does obviously not occur in post-MLF mox [6]. Therefore, the management of oxygen exposure is particularly pivotal during the PFS after MLF when most lees have been removed to make sure the level of acetaldehyde remains low.

The effective influence of oxygen on the organoleptic development of red wines has been brought into focus for many years, including enzymatic and non-enzymatic oxidations. Although the enzymatic oxidation caused by polyphenol oxidases is non-negligible during wine oxidation [7,8], such a pitfall can be avoided by using healthy grape berries uninfected by *Botrytis cinerea*. The non-enzymatic oxidation reaction mechanism of wine chemical constituents has been deeply studied and the widely accepted mechanism supports that powerful oxidant hydroxyl radicals generated from the Fenton reaction are the most immediate participants, which involves the complex reaction of oxygen, metal ions, phenols, quinones and hydrogen peroxide to finally produce acetaldehyde as the major oxidative product [9,10], while SO_2_ can be used to eliminate hydrogen peroxide to prevent the formation of hydroxyl radicals, which is an effective and low-cost additive widely used during vinification. Depending on the levels of available SO_2_, O_2_ consumption by wine contained three different patterns of chemical changes: “preSO_2_”, “normal” and “radical forming” patterns [11]. However, SO_2_ residues would have potentially negative effects on human health [12], as they can cause irritation to the mucous membranes of the nose, throat, and lungs, as well as headaches, nausea, and asthma [13]. Therefore, a reduced dosage of SO_2_ during vinification meets the future food production requirements, and the precise control of oxygen exposure is an effective means to achieve this. 

Color is one of the most important organoleptic properties of red wine, and a dramatic variation was demonstrated during wine oxidation [14], which goes along with the change of polymeric pigments [15], while phenols are generally identified as a conferrer to determine astringency, bitterness and color properties of wines [16], and they are also the major antioxidant in red wine. Recently, red wines oxidized in some consecutive air-saturation cycles were extensively characterized in order to study the kinetics of oxygen and SO_2_ consumption [17], and the evolution of color and chemical composition caused by different SO_2_-related oxidation contexts [11] or increasing air-saturation cycles [18].

However, relevant reports on the appropriate oxygen consumption during the PFS are rare. The aim of this work was to investigate the effect of different levels of oxygen exposure on acetaldehyde, color and phenols during the PFS, trying to highlight the importance of oxygen management during the PFS. The study was carried out on Cabernet Sauvignon dry red wine containing less than 10 mg/L free SO_2_ to maximally exhibit the effect of oxidation on wine. Furthermore, considering that UV–Visible spectroscopy is a more affordable and available technique, particular emphasis was placed on the changes in the color, total phenol (Folin–Ciocalteu assays), pigments (Harbertson–Adams assay), and the contribution of different anthocyanins to the overall wine color.

## 2. Results and Discussion

### 2.1. Effect of Oxygen Exposure on Dissolved Oxygen (DO), SO_2_ and Acetaldehyde during the PFS

**Figure 1:**
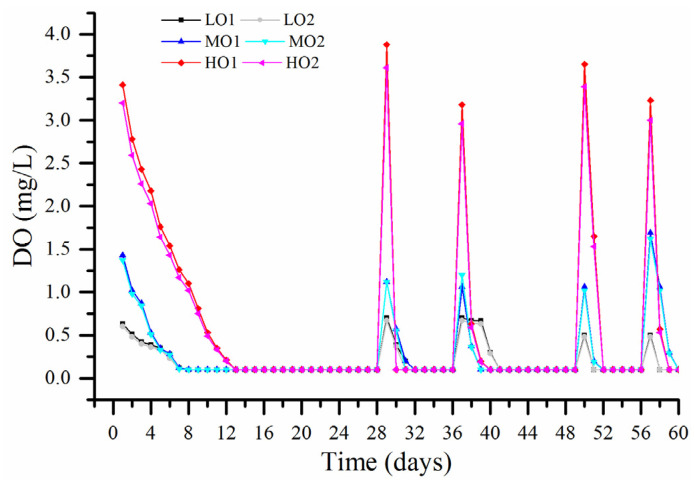
Dissolved oxygen variation for wines treated with different oxygen exposure. Abbreviations: DO, dissolved oxygen; LO, low-level oxygenation wine (0.5–0.9 mg O_2_/L per time); MO, medium-level oxygenation wine (1–1.9 mg O_2_/L per time); HO, high-level oxygenation wine (3–3.9 mg O_2_/L per time).

A comparison among the DO concentration of wines treated with different oxygen exposure during the PFS is shown in Figure 1 and Table S1 (see in the Supplementary Material), while no remarkable accumulation of dissolved oxygen was found in the control wine during the treatment. As can be deduced from the plots of each phase, the kinetics of oxygen consumption in this Cabernet Sauvignon wine were quite complex and could not be characterized as first or pseudo-first-order, and the same result was proposed for the red wine oxygen consumption in air saturation cycles [19]. Although the initial concentrations of DO were multifarious according to oxygen exposure, a general trend of oxygen consumption for all wines could be observed. The treatment of phase 1 exhibited a slower decrease in DO, reaching values below the detection threshold on the 7th and 13th day for low-level oxygenation (0.5–0.9 mg O_2_/L per time, LO); medium-level oxygenation (1–1.9 mg O_2_/L per time, MO) and high-level oxygenation (3–3.9 mg O_2_/L per time, HO) wines, respectively, in contrast to following phases of treatment that constantly exhibited a sharp decrease in DO throughout each oxygen exposure, reaching values below the detection threshold at 3 days post-treatment for all wines. 

Furthermore, the oxygen consumption rate at the beginning of each phase was the highest, especially in phases except phase 1, more than the half of initial dissolved oxygen disappeared on the first day after oxygen exposure. As previously observed by other authors [19], the initial rates of oxygen consumption in some wines and air saturations were more than 70 times higher than those observed in the immediate following measurements. This quick consumption of oxygen should be attributed to the oxidation of Fe (II) generated from the spontaneous reduction of Fe (III) at the end of each oxygenation phase when there was not enough DO. The key role of metal played in the reaction between polyphenols and oxygen was demonstrated in both synthetic wine models and real red wines [9,20].

Concentrations of free SO_2_ fell below the limit of detection (1 mg/L, verified by a previous study [21]) for HO, MO and LO wines after phases 1, 2 and 3, respectively (Table 1), which all had an initial free SO_2_ content of 5.6 ± 0.9 mg/L, suggesting that the fastest initial oxygen consumption in each following phase (phase 2–phase 5) is entirely unrelated to free SO_2_, as there was also no significant difference for bound SO_2_ in LO and MO wines before and after five phases of oxygen exposure (Table 1). The disappearance of free SO_2_ would lead to a very rapid decrease in DO in the following phases, such as phase 2 for HO, phase 3 for MO and phase 4 for LO when there was no free SO_2_. A similar relationship between DO and free SO_2_ was reported during the wine mox experiments and the chemistry of non-enzymatic wine oxidation involving hydroquinones, iron or copper catalysts, quinones, and hydroxyl radicals may explain these results [22].

**Figure 2:**
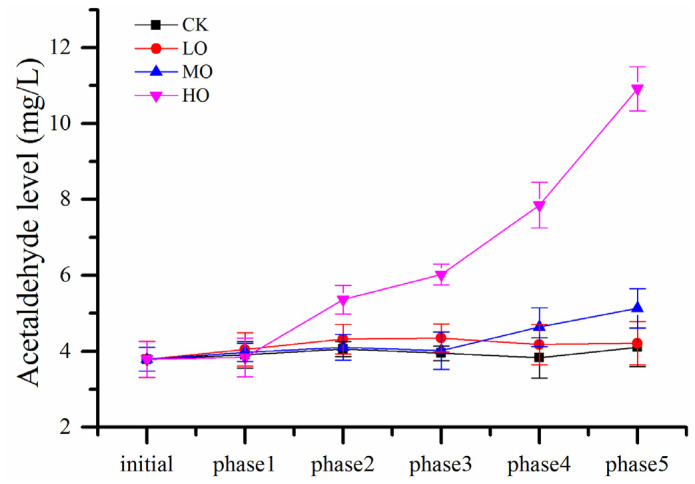
The evolution of acetaldehyde during oxygen treatment. Note: phase corresponds to Section 3.1. Abbreviations: initial, initial wine before oxidation treatment; CK, anaerobic wine (0 mg O_2_/L per time); LO, low-level oxygenation wine (0.5–0.9 mg O_2_/L per time); MO, medium-level oxygenation wine (1–1.9 mg O_2_/L per time); HO, high-level oxygenation wine (3–3.9 mg O_2_/L per time).

The level of acetaldehyde in wine was evaluated regarding sensitivity to oxygen exposure, as shown in Figure 2 and Table S2 (see in the Supplementary Material). Similar to the results of previous wine oxygenation studies, an increase in acetaldehyde level was exhibited when oxygen consumption was up to a certain degree. Acetaldehyde began to be accumulated after more than 7 mg/L of oxygen was consumed for HO wines, which no longer contained free SO_2_ at phase 2 as discussed above. This dramatic increase started contemporaneously with a much more rapid decrease in DO compared to phase 1, lasting 12 and 2 days to exhaust the dissolved oxygen at phase 1 and phase 2, respectively. This result was consistent with the study of acetaldehyde production, oxygen and SO_2_ consumption during a wine mox experiment [22] and demonstrated the important role played by free SO_2_ in regulating the generation of 1-hydroxyethyl radicals by the Fenton reaction [23]. Generally, free SO_2_ should be in the spotlight during wine production, and the suggested level was best above 5 mg/L, otherwise, a change in color would be aroused when the wine was subjected to oxygen exposure [11]. However, acetaldehyde levels were not significantly cumulative for LO and MO wines during all five oxygenation cycles although free SO_2_ was below the limit of detection using the aspiration method after phases 2 and 3. This means that the accumulation of acetaldehyde from chemical oxidation needs enough and persistent dissolved oxygen; the levels of oxygen exposure in LO and MO wines were inadequate. 

### 2.2. The Influence of Oxygen Exposure on Phenols and Color Parameters after Five Phases of Oxidation Treatments

**Figure 3:**
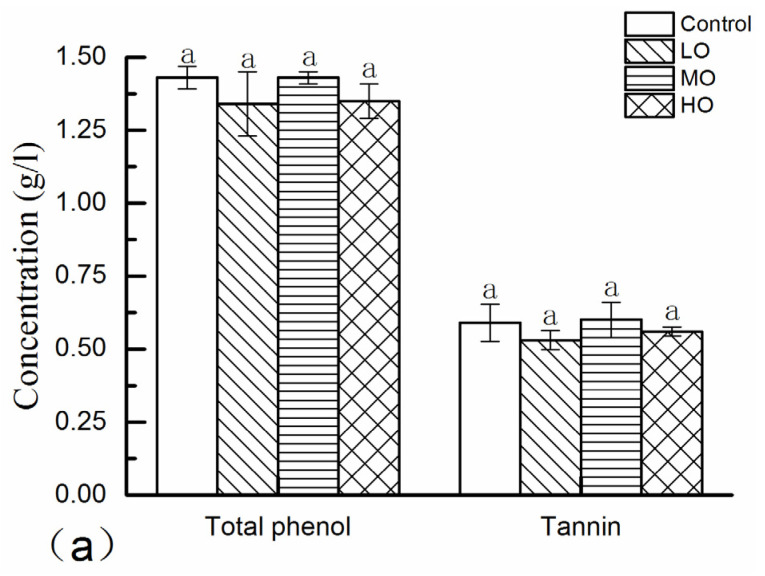

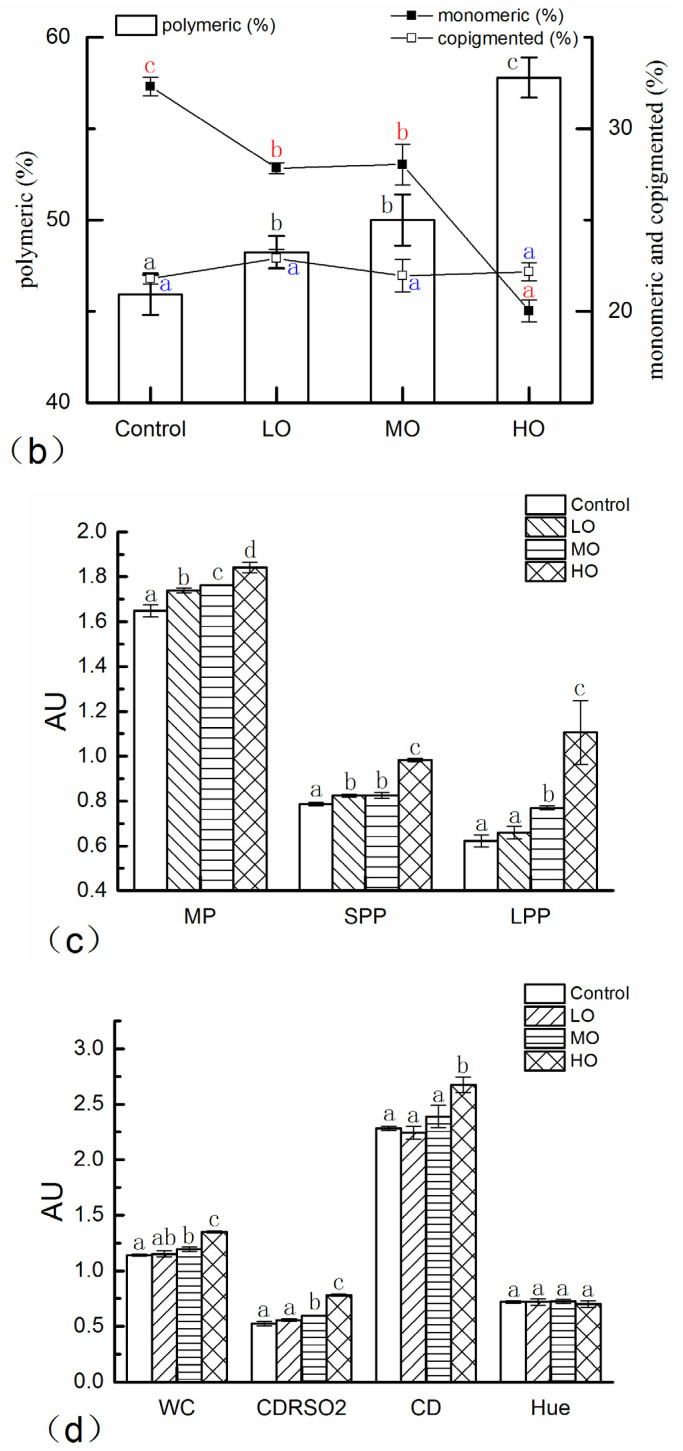
The effects of different oxygen exposure levels on the total phenol and tannin (**a**), monomeric (%), co-pigmented (%) and polymeric (%) (**b**), MP, SPP and LPP (**c**) and WC, CDRSO_2_, CD and Hue (**d**) after oxygenation of five phases. Note: Different letters (a, b, c and d) indicate significant differences at the 0.05 level. In Figure (**b**), letters of black color are for polymeric (%), letters of red color are for monomeric (%) and letters of blue color are for co-pigmented (%). Abbreviations: Control, anaerobic wine (0 mg O_2_/L per time); LO, low-level oxygenation wine (0.5–0.9 mg O_2_/L per time); MO, medium-level oxygenation wine (1–1.9 mg O_2_/L per time); HO, high-level oxygenation wine (3–3.9 mg O_2_/L per time); polymeric, polymeric anthocyanins; monomeric, monomeric anthocyanins; co-pigmented, co-pigmented anthocyanins; MP, monomeric pigments; SPP, small polymeric pigments; LPP, large polymeric pigments; CD, color density.

Phenols and color parameters of wines after five phases of oxidation treatments are shown in Figure 3 and Table S3 (see in the Supplementary Material). As shown in Figure 3a, there was no significant difference for both total phenols and tannins induced by different levels of oxygen exposure (*p* > 0.05). This expected result may indicate that polymerization reactions (anthocyanins–proanthocyanidins) rather than degradation or oxidation reactions dominated the reactions between the phenolic substances during aging or oxygenation, resulting in a marginal effect being generated on the total number of hydroxyl groups which were measured by the Folin–Ciocalteu assay [24,25,26]. The results of tannin analyzed by the BSA method also had a similar problem; some studies have shown that the tannin content of wine decreased with the aging of wine by the BSA precipitation method [27], while one study has also shown that BSA reactive tannins exhibited different trends during the oxidation process for wines with different tannin/anthocyanin ratios [28]. Because dimers and trimers did not participate in the BSA precipitation reaction, the effectiveness of the method was therefore limited to those oligomeric proanthocyanidins with a degree of polymerization greater than four units [29]. Furthermore, the larger oxidized tannin polymers might also not precipitate protein efficiently, which might be incorporated into soluble complexes with the protein [25], and these results brought out differences in tannin detection for wines with different chemical compositions. It is not clear how aging oxidation affects the equilibrium between depolymerization and condensation of tannins up to now [30]. Therefore, although the operation of total phenol analyses by Folin-assay and tannin analyses by BSA-assay are easy to operate by spectrophotometers, these two indicators cannot effectively monitor the actual state of wine oxidation aging.

Total anthocyanins in wine comprise monomeric anthocyanins, co-pigmented anthocyanins and polymeric anthocyanins. Co-pigmentation processes are most characteristic in young wines as a way to protect the free anthocyanins from oxidation reactions, while anthocyanin–cofactor complexes are weak and this conjugation reaction is reversible, resulting in these anthocyanins forming more stable color component polymeric anthocyanins during the later aging process. Recent research proved that this polymeric reaction was actually initiated during alcoholic fermentation [24]. Furthermore, polymeric anthocyanins are closely associated with “chemical age”, which is close to zero in new wine, but progressively increases with wine aging [31]. The percentages of monomeric anthocyanins (monomeric (%), co-pigmented anthocyanins (co-pigmented (%)) and polymeric anthocyanins (polymeric (%)) of wines with different oxygen exposure are shown in Figure 3b, and the results showed that there was no significant difference in co-pigmenting (%) among the wines with various oxidation treatments. Both cofactors and anthocyanins are key factors in co-pigmentation, which enhance the red color intensity of the wine. Flavonols and hydroxycinnamic acids were proved to be the two best kinds of cofactors among the groups of polyphenols in wine [24] and also had the smallest percentage loss during wine aging due to their stability and resistance to oxidation or aging compared with other groups of polyphenols [32]. Thus, this should be the one reason that co-pigmenting (%) was kept consistent after these five oxygen treatments, demonstrating the stability of anthocyanin–cofactor complexes during wine oxygenation. Although monomeric anthocyanins were the major reductive set of polyphenols during wine oxygenation [22,26,33], the main monomeric anthocyanin contained in red wines, malvidin-3-monoglucoside, had a poor correlation with the level of co-pigmentation measured in varietal wines in the previous study [34]. The reduction of co-pigmentation (%) was considerable in some research, such as the reduction of 25–43% and 34–44% in fresh Cabernet Sauvignon and Syrah wines, respectively, after 3 months of aging [34], but a reduction of only 3% was also found both in a Merlot wine and Pinot Noir wine after 4 months of aging [18]. These different results indicated that the reduction of co-pigmentation (%) depended on the chemical composition of wines or the aging conditions [24]. According to Figure 3b, monomeric (%) showed a decreasing trend with the increase in oxygenation level, the degradation rate of LO, MO and HO wines were 4.46%, 4.26% and 12.27%, respectively, compared with the control wine, and monomeric (%) of the all wines with oxidation treatment were significantly lower than that of the control wine (*p* < 0.05), but there was no significant difference between LO and MO wines (*p* > 0.05). The polymeric (%) increased with the intensification of oxygen exposure, which was contrary to the monomeric (%) and increased by 2.32%, 4.08% and 11.88% compared with control in the LO, MO and HO wines, respectively, and all the wines with oxidation treatment had significantly higher polymeric (%) than the control (*p* < 0.05), with no obvious difference between LO and MO wines (*p* > 0.05). In general, higher values of polymeric (%) and lower levels of monomeric (%) were found in wines treated with oxygen exposure, which was verified by other authors [26], and this result also applied to wine aging without human intervention. Above all, these results showed that during the PFS, the polymeric anthocyanins were primarily formed from the reaction of monomeric anthocyanins due to oxygen exposure, but the presence of cofactors prevented the co-pigmented anthocyanins having a polymerization reaction.

Wine pigments consist of monomeric pigments (MP), small polymeric pigments (SPP) and large polymeric pigments (LPP). MP is a group of compounds bleachable with sulfur dioxide, while SPP and LPP are resistant to bisulfite bleaching. However, LPP can precipitate with ovalbumin, but SPP cannot [29]. As shown in Figure 3c, both SPP and LPP were higher in the wines with higher oxygen exposure, which was in agreement with previous research, which reported that these two polymeric pigments would increase during wine aging [30] and would balance the loss of native anthocyanins and contribute to the stability of color intensity in wines. Furthermore, a greater increase in the amplitude of LPP was examined compared to SPP, which was also usually observed during wine aging [30]. Actually, the values of SPP and LPP after alcoholic fermentation were very little and they began to increase from alcoholic fermentation to bottle aging. Normally the increase in SPP was steeper than LPP at an earlier stage [29] due to the first formation of dimers, and then a trimer formed and so forth until they form polymers, and more LPP would be formed after that phase [35]. As the major pigment after alcoholic fermentation, MP also increased in the presence of oxygen, and wine with higher oxygen exposure contained a higher MP, which was consistent with previous observations [11]. Under the circumstances, the increase in MP might be due to the SO_2_ catalytic cycle and the increase in SPP and LPP was probably concerned with proanthocyanidins at a lower polymerization degree. After a long time of aging (12 months), MP was found to decrease observably [35]. Although both MP and SPP in all oxygenation wines were significantly higher than in the control wine, these two pigments and LPP were dramatically lower in the LO and MO wines compared with the HO wine, indicating that the oxygen exposure of the HO wine was too high for the wine evolution.

Similar to the chemical parameters discussed above, WC, CDRSO_2_ and CD were markedly higher in the HO wine than in other wines (*p* < 0.01), while no obvious difference was found among wines of the control, LO and MO, as shown in Figure 3d, which means that oxygen exposure of the HO wine during the PFS brought about remarkable changes in the wine organoleptic quality. The color hue, which is a ratio of the absorbance at the wavelengths 420 and 520, increased due to the important decrease in the concentration of anthocyanins (non-acylated and acylated). On the other hand, the formation of red polymeric pigments including ethylidene-bridged compounds, which contributes to the red and violet color range during wine aging, also decreases the hue value. This should be the reason why there was no marked difference among these wines after a 60-day treatment, as shown in Figure 3d, and this result was in accordance with the results of a previous wine oxygenation study [36].

The CIELab parameters of wines are depicted in Table 2. After five phases of oxygenation, the a* values were significantly higher in the HO wine, while no difference was found among the other three wines. Unlike a*, higher b*, H*ab and ΔE were more evolved when the oxygen exposure level was higher and there were significant differences between each wine. L* and C*ab values of MO and HO wines were observably different from CK and LO, and this difference was greater when the wine undergoes higher oxygen exposure. In the wine micro-oxygenation research on different pH and polyphenol level wines, H*ab revealed different evolution patterns under the influence of wine chemical characters and C* and L* trends were unclear, but only the L* value was lower in micro-oxygenated wines than their corresponding controls ignoring the effort of wine chemical characters [37]. Thus, there was no significant difference in a*, L* and C* between CK and LO, and even no difference in a* among CK, LO and MO, but all five CIELab parameters of the HO wine were dramatically different from the other wines.

Above all, the particular variation of color and phenol parameters in HO wines was accompanied by the generation of acetaldehyde. Oxygen exposure less than or equal to MO during the PFS would not cause a remarkable change in color and phenol parameter characteristics like with HO wines without the protection of free SO_2_. Nevertheless, similar but less significant changes in some chemical indexes (MP, SPP, LPP and WC, etc.) induced by low-level or medium-level oxygenation were also found with no acetaldehyde accumulation in LO and MO wines; it is unclear if acetaldehyde plays an important role in these variations. There are two hypotheses, one is that a small quantity of acetaldehyde is generated during oxygen exposure of LO and MO wines, but it is not enhancive due to its quite strong chemical reaction capacity, and the other is that no acetaldehyde is produced under the weak oxidation environment. Further validation and expedition are needed because this is a critical point in explaining the essence of chemical evolution during red wine aging.

Here, it is worth noting that all parameters affecting phenolic composition will certainly influence the oxygen consumption effect as previously observed by other authors [19,28], and different wines should suffer from different levels of oxygen exposure. More emphasis also needs to be put on the research about how to predict the oxygen resistance ability of dry red wine before the PFS, which will benefit in elaborating the oxygen management plan in the absence of free SO_2_ during the PFS.

### 2.3. Evolution of Critical Wine Chemical Parameters during Five Phases of Oxidation Treatments

Pearson correlation coefficients were further applied in order to evaluate the correlations between oxygen consumption and each selected chemical parameter before and after each oxygenation cycle, which are shown in Table 3. All the parameters were significantly associated with oxygen consumption except tannin, Hue, co-pigmentation (%) and a*, meanwhile, there were also no significant differences among CK, LO, MO and HO after five cycles for these four parameters as discussed above (Table 2 and Figure 3). Furthermore, 12 parameters had values of *r* above 0.85, in which only monomeric (%) and L* were negatively correlated to oxygen consumption, and similar correlations for monomeric (%) and L* were previously reported [26,33,37].

**Figure 4:**
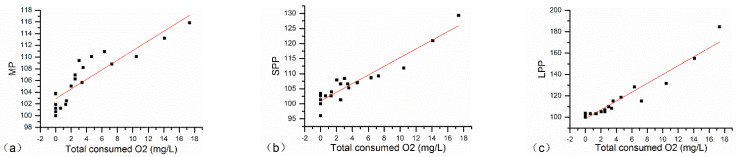

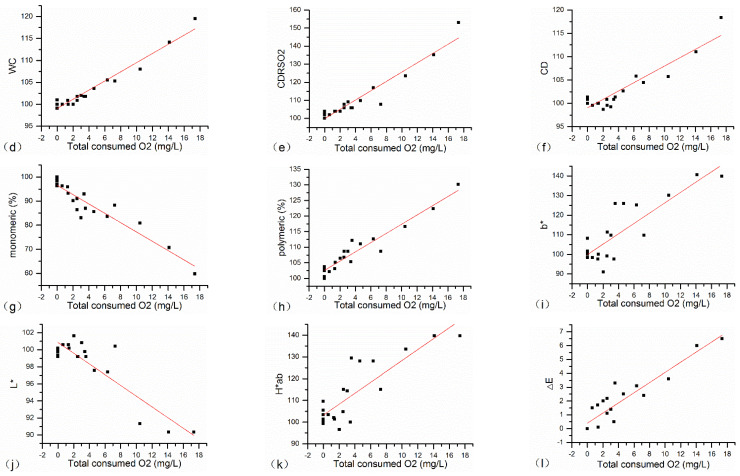
Plots showing the evolution with the amount of oxygen consumed of MP (**a**), SPP (**b**), LPP (**c**), WC (**d**), CDRSO_2_ (**e**), CD (**f**), monomeric (%) (**g**), polymeric (%) (**h**), b* (**i**), L* (**j**), H*ab (**k**), and ΔE (**l**) of all the wines before and after each oxygenation cycle. In all cases, initial values were normalized to 100% by initial values. Abbreviations: MP, monomeric pigments; SPP, small polymeric pigments; LPP, large polymeric pigments; WC, total color of free anthocyanins and anthocyanins eventually involved in bisulfite adducts; CDRSO_2_, color due to derivatives resistant to SO_2_ bleaching; CD, color density; polymeric, polymeric anthocyanins; monomeric, monomeric anthocyanins; co-pigmented, co-pigmented anthocyanins.

The 12 parameters highly correlated with oxygen consumption during the five oxidation cycles were normalized by initial values and represented vs. oxygen consumption for all the wines before and after each oxygenation cycle, as shown in Figure 4 (Table S4, see in the Supplementary Material). Numerical results such as slope, ±Standard Deviation, and change per mg/L of consumed O_2_ of each parameter are summarized in Table 4. As two kinds of polymeric pigments in wines determined by the Harbertson–Adams assay, LPP is a class of pigments that precipitate with protein, while SPP is a class of pigments that do not precipitate. The results showed that LPP increased much faster than SPP when 1 mg/L oxygen was consumed by the wine, and the increasing rate of LPP (4.86%) was even treble that of LPP (1.69%). Similar trends happened during winemaking and aging, as a greater formation of LPP compared to SPP was usually observed [30]. It is worth mentioning that the change of LPP was most significant among the selected chemical parameters, followed closely by CDRSO_2_, which was 3.06% per mg/L of consumed O_2_. These all made perfect sense in that the formation of polymeric pigments might balance the loss of native anthocyanins contributing to the color stability of wines, such as one of the most important attributes of higher SO_2_-resistant ability [38], at the same time, monomeric (%) decreased 2.32% per mg/L of consumed O_2,_ proving the loss of native anthocyanins. For CIELab parameters, b* and H*ab increased by around 2.3%, while L* decreased slightly (−0.56% on average per mg/L of Oxygen consumed).

**Figure 5:**
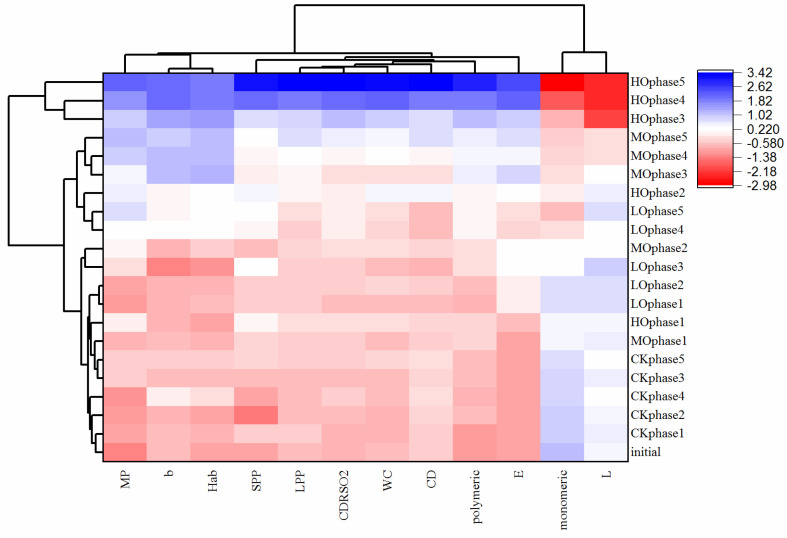
Clustered heatmap of the evolution of selected chemical parameters during the oxygenation cycles. Abbreviations: initial, initial wine before oxidation treatment; CK, anaerobic wine (0 mg O_2_/L per time); LO, low-level oxygenation wine (0.5–0.9 mg O_2_/L per time); MO, medium-level oxygenation wine (1–1.9 mg O_2_/L per time); HO, high-level oxygenation wine (3–3.9 mg O_2_/L per time); MP, monomeric pigments; SPP, small polymeric pigments; LPP, large polymeric pigments; WC, total color of free anthocyanins and anthocyanins eventually involved in bisulfite adducts; CDRSO_2_, color due to derivatives resistant to SO_2_ bleaching; CD, color density; polymeric, polymeric anthocyanins; monomeric, monomeric anthocyanins; co-pigmented, co-pigmented anthocyanins.

**Figure 6:**
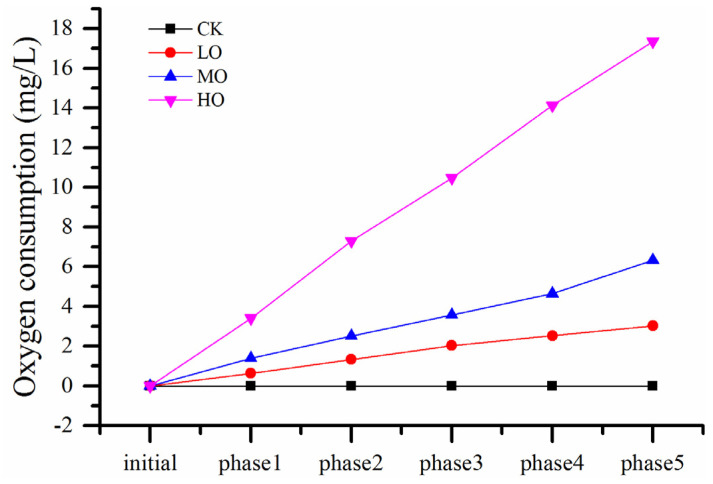
Total oxygen consumption during treatment. Abbreviations: initial, initial wine before oxidation treatment; CK, anaerobic wine (0 mg O_2_/L per time); LO, low-level oxygenation wine (0.5–0.9 mg O_2_/L per time); MO, medium-level oxygenation wine (1–1.9 mg O_2_/L per time); HO, high-level oxygenation wine (3–3.9 mg O_2_/L per time).

The evolution of 12 selected chemical parameters was also visualized in the clustered heatmap, as shown in Figure 5 (Table S4, see in the Supplementary Material). All the wines at each oxygenation cycle consumed different levels of oxygen and could be easily classified into three distinct clusters which resolved with unique features of the components in each. Generally, wines in cluster 1 were the highest oxygenation wines (HOphase3, HOphase4 and HOphase5) with the highest levels of MP, SPP, LPP, WC, CDRSO_2_, CD, polymeric (%), b*, H*ab and ΔE, and lowest levels of monomeric (%) and CIELab parameter L, which consumed above 10 mg/L oxyge, n as shown in Figure 6. Wines in cluster 3 were the lowest oxygenation wines (HOphase1, MOphase2, MOphase1, LOphase3, LOphase2, LOphase1 and all the CK wines), which consumed under 3 mg/L oxygen, as shown in Figure 6, showing completely opposite characteristics compared to wines in cluster 1. Cluster 2 was comprised of wines that consumed oxygen between 3 and 10 mg/L, which had moderate chemical characteristics. According to the clustered heatmap, wines were classified based on the levels of oxygen consumed, and the impact of different cycles was minimal although five oxygenation cycles continued for 60 days.

Because of the successful classification of different wines according to the levels of oxygen consumption, a PLSR model explaining oxygen consumption at each oxygenation cycle interval was also built using the selected 12 parameters, which had the values of *r* above 0.85 shown in Table 3:

oxygen consumption (mg/L) = −32.97 + 5.67 × MP + 7.61 × SPP + 3.48 × LPP + 7.54 × WC + 0.21 × b − 0.24 × L + 0.184 × H_ab_ + 0.22 × ΔE + 6.53 × CDRSO_2_ + 3.98 × CD − 0.13 × Monomeric (%) + 0.13 × polymeric (%)

where b was b*; L was L*; and Hab was H*ab. The regression model behaved fairly well in the range of oxygen consumption explored, giving good regression coefficients of 0.957 for the first component and 0.967 for the first two, meanwhile the model with one PC explained 89.7% of the original variance. Variable importance in the projection (VIP) of the 12 independent variables were all above 1, except MP, b*, L*, and H*ab with the VIP values between 0.92 and 1. The model confirmed the behavior that was already reflected in the exploratory study, in which oxygen consumption was positively related to MP, SPP, LPP, WC, CDRSO_2_, CD, polymeric (%), b*, H*ab and ΔE, and negatively correlated to the monomeric (%) and CIELab parameter L*.

Most noteworthy was that the trend of MP ran counter to that of monomeric (%) along with wine oxidation according to the PLSR model and Figure 4, while both two chemical parameters may represent monomeric pigments in the wine. Actually, although MP, SPP and LPP were all increasing with the incremental amount of oxygen consumed, the trend-line slope of MP was much less than that of SPP and LPP, 0.82, 1.4 and 4.13, respectively, as shown in Table 4, which means polymeric pigments aggrandized much faster, especially LPP. Therefore, the increase in polymeric (%), which was interrelated with polymeric pigments, consequentially resulted in the decrease in monomeric (%).

## 3. Materials and Methods

### 3.1. Wines and Oxygenation Trials

The wine fermentation was performed at the Chateau Changyu Baron Balboa, Xinjiang, China on healthy grape berries, *Vitis vinifera* cv. Cabernet Sauvignon with 23.5 °B during the 2017 harvest season. The wine was prepared with standard winemaking techniques using the *Saccharomyces cerevisiae* yeast Lalvin^®^ RC212 (Lallemand, Blagnac Cedex, France). After the completion of spontaneous malolactic fermentation (MLF), the wines received an addition of potassium metabisulfite to result in a final concentration of free SO_2_ of 26 mg/L and matured in the stainless steel tanks for 3 months, and then the wine was separated into one stainless steel tanks of 200 L with N_2_ protection for full-tank aging at 4 ± 1 °C refrigeration storage for another 3 months of cold settling to remove lees further, and 180 L supernatant wine would be used in the following experiment. The base parameters (mean ± standard deviation) of wine before the oxygenation experiment were: ethanol content 13.30 ± 0.11% *v*/*v*, pH 3.71 ± 0.02, titratable acidity 5.61 ± 0.09 g/L expressed as tartaric acid, residual sugars 0.40 ± 0.02 g/L, volatile acidity 0.48 ± 0.01 g/L, free SO_2_ 5.6 ± 0.9 mg/L and bound SO_2_ 28.9 ± 2.2 mg/L. Then, the wine was divided into eight stainless steel tanks of 22 L each configured with a PreSens PST3 oxygen sensor (Nomacorc LLC, Zebulon, NC, USA), which was similar to the tanks used in the previous study [3,22].

The oxygen exposure treatments consisted of a control (no oxygenation), low-level oxygenation (LO, 0.5–0.9 mg O_2_/L per time), medium-level oxygenation (MO, 1–1.9 mg O_2_/L per time) and high-level oxygenation (HO, 3–3.9 mg O_2_/L per time) (Figure 6 and Table S5, see in the Supplementary Material), each treatment was conducted in duplicate. The oxygen was supplied to the wine liquid by plastic tubes with 2 mm inner diameter delivered by the pure oxygen cylinder at high pressure to insure dissolved oxygen (DO) of each wine reached the target value, accompanied by analyzing with a Nomasense oxygen analyzer (Nomacorc SA, Thimister Clermont, Belgium). Then, oxygen input was terminated by closing oxygen valve of each tank when DO reach the target value, while N_2_ was used to empty oxygen of the tank headspace after each oxygen delivery. Each tank was stirred using magnetic stir bars during oxygen input to ensure that the oxygen was thoroughly mixed into the wine as diffusion occurred. After that, all the wines were stored in the same room at a constant temperature (18–20 °C), in ambient air. When all dissolved oxygen in tanks was below 0.1 mg/L, this anaerobic environment went on for several days depending on actual process requirements before the next oxygen carriage, and this process was repeated five times to simulate clarification (phase 1), fining (phase 2), cold stabilization (phase 3), filtration (phase 4) and bottling (phase 5) respectively. Total oxygen consumption at each phase of each wine is shown in Figure 6 and Table S5.

Wines were analyzed in triplicate before oxygen exposure treatment. Then, the wine of each tank was sampled after each round of oxygen exposure for analyses in duplicate. Subsamples were stored in small containers with N_2_ protection in the dark at 4 °C. All analyses were carried out within 48 h.

### 3.2. Sulfur Dioxide and Acetaldehyde Determination

The concentration of free and total SO_2_ was analyzed using the aspiration/titration method [39]. The free SO_2_ was removed from the wine by passing a stream of air through the acidified sample, which was then absorbed by a hydrogen peroxide/mixed indicator solution, and the formed sulfuric acid was then titrated with standard sodium hydroxide. After that, strong acidic conditions and heat dissociated the bound SO_2_ complex, the released SO_2_ was then trapped and determined as described above for free SO_2_.

For the analysis of acetaldehyde, 2,4-dinitrophenylhy-drazine (DNPH) derivatization method was used [40]. A total of 100 μL wine was dispensed to a 2 mL vial, followed by 20 μL of freshly prepared 1120 mg/L SO_2_ solution, and then 20 μL of 25% sulfuric acid (*v*/*v*) was added, followed by 140 μL of 8 g/L DNPH reagent. The solution was allowed to react for 15 min at 65 °C and then promptly cooled to room temperature. Completely derivatized reaction solution was analyzed by Waters e2695 HPLC (Milford, MA, USA) equipped with a 2998 diode array detector after it was filtered by 0.20 μm MicroLiter PTFE membrane filters (Jinteng Experimental Equipment Co., Ltd., Tianjin, China). Separation occurred on An Agilent ZORBAX Rapid Resolution HT, SB-C18 column (1.8 μm, 4.6 × 100 mm) held at 35 °C with a flow rate of 0.75 mL/min. The analysis was quantified at 365 nm using external calibration standards with linear regression analysis. The identification of acetaldehyde-DNPH was made by comparison with retention times and chromatographic profile reported by Han et al. [40]. Data analysis and peak integration were carried out using Empower 3 chromatography workstation.

### 3.3. Color Analyses

Chromatic characteristics and spectrophotometric measures were determined using an Agilent Cary 60 UV–Vis spectrophotometer (Agilent Technologies). Optical density of undiluted wine filtered by 0.20 µm MicroLiter PTFE membrane filters was measured at 420, 520, and 620 nm, using a 1 mm optical path glass cell. Colorimetric calculations were performed according to the formulas proposed by Glories [41]:

Color Density (CD) = A420 + A520 + A620; Hue = A420/A520

CIELab parameters were measured in terms of D65illuminant and 10° observer and expressed in terms of L*, a*, b*, the parameter C*_ab_ calculated as (a*^2^ + b*^2^)^1/2^, H*_ab_ calculated as tan^−1^(b*/a*), ΔE calculated as (ΔL*^2^ + Δa*^2^ + Δb*^2^)^1/2^ [42].

### 3.4. Spectrophotometric Measurements of Total Phenol, Pigments and Co-pigmented Anthocyanins

The Folin–Ciocalteu method was used for the quantification of total phenol after dilution of samples [43]. The results were expressed as gallic acid equivalents, through construction of a standard curve. The procedure for the determination of total tannin, monomeric pigments (MP), small polymeric pigments (SPP) and large polymeric pigments (LPP) was based on the procedure developed by Habertson, Picciotto, and Adams [29].

The contribution of the monomeric (non-co-pigmented), co-pigmented and polymeric anthocyanins to the total wine color was determined following the described method [24]. The wine sample was adjusted to pH 3.6 and membrane filtered by 0.45 µm MicroLiter PTFE membrane filters. To the first 2 mL of wine, 20 μL of 20% (*w*/*v*) acetaldehyde was added and the sample was allowed to stand for approximately 45 min; to another 2 mL of wine, 160 μL of 5% (*w*/*v*) SO_2_ was added and measured after10 min. The absorbance of these two samples was measured at 520 nm in a 10 mm glass cuvette, and the respective measures were named WC (total color of free anthocyanins and anthocyanins eventually involved in bisulfite adducts) and CDRSO_2_ (color due to derivatives resistant to SO_2_ bleaching). Moreover, 100 μL of wine sample was placed into 1900 μL of bitartrate buffer and measured at 520 nm in a 10 mm glass cuvette, A_520_^wine^ reading was corrected for the dilution by multiplying by 20. The following equations were used to calculate the percentage of each fraction to color contribution:

(1) monomeric (%) = (A_520_^wine^ – CDRSO_2_) × 100/WC;

(2) co-pigmented (%) = (WC – A_520_^wine^) × 100/WC;

(3) polymeric (%) = CDRSO_2_*100/WC.

### 3.5. Statistical Analysis

Analysis of variance (ANOVA) and Duncan’s multiple range test (*p* < 0.05) were carried out using SPSS 18.0 software. All data were means of four values (2 experimental replicates × 2 analytical replicates). Partial least square regression (PLSR) was carried out using the PLSR module of the IBM SPSS Statistics 25.

## 4. Conclusions

This study provided clear evidence of a strong link between oxygen exposure and wine characteristics (color and phenols) evolution during the PFS and demonstrated the significance of the precise control of oxygen during PFS operations. Therefore, scientific oxygen management to modulate dry red wine evolution during the PFS may be an interesting complement to the dose reduction of SO_2_. Furthermore, our results suggest that acetaldehyde may be not considered an accurate indicator of oxygenation degree for red wine because some chemical indexes were hard to keep unchanged with no acetaldehyde accumulation during low oxygenation treatment. Twelve chemical parameters of phenols and color parameters were selected to effectively monitor wine oxidation status, which were used as variables in a successful PLSR model to explain the oxygen consumption of the wine. In the future, it would be worthwhile to trace the oxygen consumption during the PFS using the model and to accurately predict the aging potential of wine according to the evolution of the above chemical parameters.

## Figures and Tables

**Table 1:** Content of free and bound SO_2_ of wines treated with different oxygen exposures.

Parameter	Oxygen Level	Initial	After Phase 1	After Phase 2	After Phase 3	After Phase 5
Free SO_2_ (mg/L)	Control	5.6 ± 0.9	5.5 ± 0.6	NT	NT	5.2 ± 1.1
	LO	5.6 ± 0.9	4.3 ± 0.2	2.6 ± 0.5	0	NT
	MO	5.6 ± 0.9	3.3 ± 0.5	0	NT	NT
	HO	5.6 ± 0.9	0	NT	NT	NT
Bound SO_2_ (mg/L)	Control	28.9 ± 2.2	NT	NT	NT	27.9 ± 3.1
	LO	28.9 ± 2.2	NT	NT	NT	25.4 ± 2.7
	MO	28.9 ± 2.2	NT	NT	NT	24.7 ± 3.3
	HO	28.9 ± 2.2	NT	NT	NT	21.5 ± 2.9

Abbreviations: NT, no test; LO, low-level oxygenation wine (0.5–0.9 mg O_2_/L per time); MO, medium-level oxygenation wine (1–1.9 mg O_2_/L per time); HO, high-level oxygenation wine (3–3.9 mg O_2_/L per time).

**Table 2:** The effects of different oxygen exposure levels on CIELab parameters after oxygenation of five phases.

CIELab	Control	LO	MO	HO
a*	45.5 ± 0.3 ^a^	45.3 ± 0.3 ^a^	45.6 ± 0.3 ^a^	46.4 ± 0.3 ^b^
b*	12.5 ± 0.3 ^a^	13.5 ± 0.3 ^b^	15.4 ± 0.3 ^c^	17.2 ± 0.3 ^d^
L*	49.3 ± 0.4 ^c^	50.1 ± 0.5 ^c^	48.3 ± 0.5 ^b^	44.9 ± 0.5 ^a^
C*_a,b_	47.1 ± 0.4 ^a^	46.8 ± 0.4 ^a^	48.1 ± 0.4 ^b^	49.5 ± 0.3 ^c^
H*_a,b_	15.4 ± 0.2 ^a^	16.7 ± 0.2 ^b^	18.7 ± 0.2 ^c^	20.3 ± 0.2 ^d^
ΔE	0 ^a^	1.4 ± 0.001 ^b^	3.0 ± 0.02 ^c^	6.5 ± 0.01 ^d^

Note: Different letters (^a^, ^b^, ^c^ and ^d^) indicate significant differences at the 0.05 level. Abbreviations: Control, anaerobic wine (0 mg O_2_/L per time); LO, low-level oxygenation wine (0.5–0.9 mg O_2_/L per time); MO, medium-level oxygenation wine (1–1.9 mg O_2_/L per time); HO, high-level oxygenation wine (3–3.9 mg O_2_/L per time).

**Table 3:** Descriptive statistics and Pearson correlation coefficient *r* versus oxygen consumption (OC), acetaldehyde (AD) or phase (PS) of the variables studied.

Variable	Min	Max	Mean	SD	*r* (OC)	*r* (AD)	*r* (PS)
TP (mg/L)	1342.44	1501.12	1420.51	41.21	−0.460 *	−0.471 *	−0.548 *
Tannin (mg/L)	533.87	645.66	601.81	28.01	ns	ns	−0.582 **
MP	1.59	1.84	1.69	0.071	0.891 **	0.768 **	0.631 **
SPP	0.73	0.98	0.81	0.056	0.945 **	0.930 **	0.491 *
LPP	0.60	1.11	0.69	0.12	0.962 **	0.975 **	0.483 *
WC	1.12	1.35	1.16	0.058	0.979 **	0.975 **	0.464 *
CDRSO_2_	0.51	0.78	0.56	0.066	0.960 **	0.973 **	0.510 *
CD	2.23	2.68	2.32	0.11	0.929 **	0.971 **	0.441 *
Hue	0.69	0.73	0.71	0.0098	ns	ns	ns
monomeric (%)	20.03	33.52	29.89	3.33	−0.946 **	−0.903 **	−0.593 **
co-pigmented (%)	21.01	22.9	21.99	0.49	ns	ns	ns
polymeric (%)	44.40	57.80	48.07	3.30	0.962 **	0.914 **	0.562 **
a*	44.50	46.70	45.66	0.69	ns	ns	ns
b*	11.20	17.30	13.54	1.84	0.853 **	0.754 **	0.583 **
L*	44.80	50.40	48.83	1.69	−0.895 **	−0.858 **	ns
C* _ab_	46.00	49.70	47.73	1.00	0.607 **	0.604 **	ns
H* _ab_	14.10	20.40	16.48	2.09	0.851 **	0.735 **	0.612 **
ΔE	0	6.50	1.80	1.91	0.930 **	0.861 **	0.446 *

Note: phase included phases 1–5, which correspond to Section 3.1. Abbreviations: TP, total phenol (Folin–Ciocalteu assays), which were expressed as gallic acid equivalents; MP, monomeric pigments; SPP, small polymeric pigments; LPP, large polymeric pigments; WC, total color of free anthocyanins and anthocyanins eventually involved in bisulfite adducts; CDRSO_2_, color due to derivatives resistant to SO_2_ bleaching; CD, color density; polymeric, polymeric anthocyanins; monomeric, monomeric anthocyanins; co-pigmented, co-pigmented anthocyanins; * *p* < 0.05; ** *p* < 0.01; ns, not significant.

**Table 4:** Average rates of change of pigments and color during five-phase oxidation.

Parameter	Slope	±Standard Deviation	Change per mg/L of Consumed O_2_
MP	0.82	0.096	0.91%
SPP	1.44	0.11	1.69%
LPP	4.13	0.27	4.86%
WC	1.04	0.050	1.12%
CDRSO_2_	2.58	0.17	3.06%
CD	0.89	0.081	1.06%
monomeric (%)	−1.94	0.15	−2.32%
polymeric (%)	1.48	0.096	1.74%
b*	2.64	0.37	2.30%
L*	−0.63	0.072	−0.56%
H*_ab_	2.51	0.36	2.29%
ΔE ^†^	0.37	0.033	0.37

Abbreviations: MP, monomeric pigments; SPP, small polymeric pigments; LPP, large polymeric pigments; WC, total color of free anthocyanins and anthocyanins eventually involved in bisulfite adducts; CDRSO_2_, color due to derivatives resistant to SO_2_ bleaching; CD, color density; polymeric, polymeric anthocyanins; monomeric, monomeric anthocyanins; co-pigmented, co-pigmented anthocyanins; ^†^, the actual value without normalization.

## Data Availability

The study did not report any data.

## Supplementary Materials

The following supporting information can be downloaded at: https://www.mdpi.com/article/10.3390/molecules27196692/s1, Table S1: Dissolved oxygen (mg/L) variation for wines treated with different oxygen exposure; Table S2: The evolution of acetaldehyde (mg/L) during oxygen treatment; Table S3: The effects of different oxygen exposure levels on the total phenol and tannin, monomeric (%), co-pigmented (%) and polymeric (%), MP, SPP and LPP and WC, CDRSO_2_, CD and Hue after oxygenation of five phases; Table S4: The evolution of selected chemical parameters during the five oxygenation cycles; Table S5: Total oxygen consumption (mg/L) during treatment.

## Sample Availability

Samples of the compounds are not available from the authors.
