# Supplementary material for: Influence of Oxygen Management during the Post-Fermentation Stage on Acetaldehyde, Color, and Phenolics of Vitis vinifera L. Cv. Cabernet Sauvignon Wine
Source: Molecules. 2022 Oct 8;27(19):6692. doi: 10.3390/molecules27196692 (PMC9572646; doi:10.3390/molecules27196692)
Supplement: Supplementary file 1 [file molecules-27-06692-s001.zip › molecules-1948582-supplementary.pdf]

Influence of Oxygen Management during the Post-Fermentation Stage on Acetaldehyde, Color, and Phenolics of *Vitis vinifera* L. Cv. Cabernet Sauvignon Wine

Lingmin Dai [1], Yuhang Sun [1], Muqing Liu [1], Xiaoqian Cui [1], Jiaqi Wang [1], Jiming Li [2] and Guomin Han [1,*]

[1] School of Bioengineering, Qilu University of Technology (Shandong Academy of Sciences), Jinan 250353, China

[2] Yantai Changyu Group Corporation Ltd., Yantai 264001, China

* Correspondence: gmhan@qlu.edu.cn

Table S1. Dissolved oxygen (mg/L) variation for wines treated with different oxygen exposure
Table S2. The evolution of acetaldehyde (mg/L) during oxygen treatment
Table S3. The effects of different oxygen exposure levels on the total phenol and tannin, monomeric (%), copigmented (%) and polymeric (%), MP, SPP and LPP and WC, CDRSO2, CD and Hue after oxygenation of five phases
Table S4. The evolution of selected chemical parameters during the five oxygenation cycles
Table S5. Total oxygen consumption (mg/L) during treatment

Table S1. Dissolved oxygen (mg/L) variation for wines treated with different oxygen exposure.

Time (d)	LO1	LO2	MO1	MO2	HO1	HO2
1	0.63	0.6	1.43	1.37	3.41	3.2
2	0.51	0.48	1.02	0.98	2.78	2.59
3	0.42	0.4	0.87	0.84	2.43	2.26
4	0.39	0.36	0.53	0.51	2.18	2.03
5	0.35	0.33	0.35	0.33	1.76	1.64
6	0.28	0.23	0.28	0.27	1.54	1.43
7	0.12	0.11	0.12	0.11	1.26	1.17
8	0.1	0.1	0.1	0.1	1.1	1.02
9	0.1	0.1	0.1	0.1	0.81	0.75
10	0.1	0.1	0.1	0.1	0.53	0.49
11	0.1	0.1	0.1	0.1	0.35	0.33
12	0.1	0.1	0.1	0.1	0.21	0.2
13	0.1	0.1	0.1	0.1	0.1	0.1
14	0.1	0.1	0.1	0.1	0.1	0.1
15	0.1	0.1	0.1	0.1	0.1	0.1
16	0.1	0.1	0.1	0.1	0.1	0.1
17	0.1	0.1	0.1	0.1	0.1	0.1
18	0.1	0.1	0.1	0.1	0.1	0.1
19	0.1	0.1	0.1	0.1	0.1	0.1
20	0.1	0.1	0.1	0.1	0.1	0.1
21	0.1	0.1	0.1	0.1	0.1	0.1
22	0.1	0.1	0.1	0.1	0.1	0.1
23	0.1	0.1	0.1	0.1	0.1	0.1
24	0.1	0.1	0.1	0.1	0.1	0.1
25	0.1	0.1	0.1	0.1	0.1	0.1
26	0.1	0.1	0.1	0.1	0.1	0.1
27	0.1	0.1	0.1	0.1	0.1	0.1
28	0.1	0.1	0.1	0.1	0.1	0.1
29	0.7	0.67	1.12	1.12	3.88	3.61
30	0.39	0.37	0.57	0.55	0.1	0.1
31	0.2	0.1	0.2	0.1	0.1	0.1
32	0.1	0.1	0.1	0.1	0.1	0.1
33	0.1	0.1	0.1	0.1	0.1	0.1
34	0.1	0.1	0.1	0.1	0.1	0.1
35	0.1	0.1	0.1	0.1	0.1	0.1
36	0.1	0.1	0.1	0.1	0.1	0.1
37	0.7	0.67	1.06	1.2	3.18	2.96
38	0.67	0.64	0.37	0.36	0.63	0.59
39	0.67	0.63	0.1	0.1	0.2	0.19
40	0.3	0.29	0.1	0.1	0.1	0.1
41	0.1	0.1	0.1	0.1	0.1	0.1
42	0.1	0.1	0.1	0.1	0.1	0.1
43	0.1	0.1	0.1	0.1	0.1	0.1
44	0.1	0.1	0.1	0.1	0.1	0.1
45	0.1	0.1	0.1	0.1	0.1	0.1
46	0.1	0.1	0.1	0.1	0.1	0.1
47	0.1	0.1	0.1	0.1	0.1	0.1
48	0.1	0.1	0.1	0.1	0.1	0.1
49	0.1	0.1	0.1	0.1	0.1	0.1
50	0.5	0.48	1.06	1.02	3.65	3.39
51	0.1	0.1	0.2	0.19	1.65	1.53
52	0.1	0.1	0.1	0.1	0.1	0.1
53	0.1	0.1	0.1	0.1	0.1	0.1
54	0.1	0.1	0.1	0.1	0.1	0.1

55	0.1	0.1	0.1	0.1	0.1	0.1
56	0.1	0.1	0.1	0.1	0.1	0.1
57	0.5	0.48	1.69	1.62	3.23	3
58	0.1	0.1	1.06	1.02	0.57	0.53
59	0.1	0.1	0.3	0.29	0.1	0.1
60	0.1	0.1	0.1	0.1	0.1	0.1

Abbreviations: LO, low-level oxygenation wine (0.5–0.9 mg O_2/L per time); MO, medium-level oxygenation wine (1–1.9 mg O_2/L per time); HO, high-level oxygenation wine (3–3.9 mg O_2/L per time).

Table S2. The evolution of acetaldehyde (mg/L) during oxygen treatment.

	CK	LO	MO	HO
initial	3.79±0.31	3.79±0.48	3.79±0.31	3.79±0.47
phase1	3.91±0.35	4.05±0.44	3.97±0.24	3.84±0.51
phase2	4.06±0.2	4.32±0.39	4.11±0.34	5.36±0.38
phase3	3.95±0.19	4.35±0.37	4.02±0.49	6.02±0.27
phase4	3.83±0.53	4.18±0.53	4.64±0.51	7.85±0.6
phase5	4.11±0.51	4.21±0.57	5.13±0.52	10.92±0.58

Abbreviations: initial, initial wine before oxidation treatment; CK, anaerobic wine (0 mg O_2/L per time); LO, low-level oxygenation wine (0.5–0.9 mg O_2/L per time); MO, medium-level oxygenation wine (1–1.9 mg O_2/L per time); HO, high-level oxygenation wine (3–3.9 mg O_2/L per time).

Table S3. The effects of different oxygen exposure levels on the total phenol and tannin, monomeric (%), copigmented (%) and polymeric (%), MP, SPP and LPP and WC, CDRSO2, CD and Hue after oxygenation of five phases

	Control	LO	MO	HO
Total phenol	1.43±0.039	1.34±0.11	1.43±0.02	1.35±0.06
Tannin	0.59±0.064	0.530.033	0.6±0.061	0.56±0.015
monomeric (%)	32.3±0.5	27.84±0.31	28.04±1.12	20.03±0.58
copigmented (%)	21.8±0.32	22.9±0.5	21.96±0.89	22.17±0.52
polymeric (%)	45.92±1.11	48.24±0.89	50.00±1.40	57.80±1.08
MP	1.65±0.028	1.74±0.011	1.76±0.002	1.84±0.023
SPP	0.79±0.0075	0.82±0.006	0.83±0.013	0.98±0.007
LPP	0.62±0.027	0.66±0.029	0.77±0.0085	1.11±0.14
WC	1.14±0.01	1.15±0.03	1.19±0.02	1.35±0.01
CDRSO2	0.52±0.02	0.56±0.01	0.60±0.0014	0.78±0.01
CD	2.28±0.02	2.24±0.06	2.39±0.1	2.68±0.07
Hue	0.72±0.01	0.72±0.03	0.72±0.02	0.70±0.032

Abbreviations: Control, anaerobic wine (0 mg O_2/L per time); LO, low-level oxygenation wine (0.5–0.9 mg O_2/L per time); MO, medium-level oxygenation wine (1–1.9 mg O_2/L per time); HO, high-level oxygenation wine (3–3.9 mg O_2/L per time); polymeric, polymeric anthocyanins; monomeric, monomeric anthocyanins; copigmented, copigmented anthocyanins; MP, monomeric pigments; SPP, small polymeric pigments; LPP, large polymeric pigments; CD, color density.

Table S4. The evolution of selected chemical parameters (means of four values) during the oxygenation cycles

Wine	TCO (mg/L)	TP (mg/L)	Tannin (mg/L)	MP	SPP	LPP	WC	$CDRSO_2$	CD	Hue	Monomeric (%)	Copigmented (%)	Polymeric (%)	a*	b*	L*	C*ab	H*ab	E
initial	0	1438.56	611.51	1.59	0.76	0.6	1.13	0.51	2.26	0.7	33.52	22.08	44.4	46.5	12.3	49.6	48.1	14.6	0
CKphase1	0	1400.77	595.6779	1.62	0.78	0.62	1.12	0.51	2.26	0.69	33.17	22.15	44.68	46.5	12.3	49.7	48.1	14.8	0
CKphase2	0	1482.21	613.9915	1.61	0.73	0.61	1.12	0.52	2.28	0.72	32.94	21.01	46.05	46.7	12.1	49.5	48.2	14.5	0
CKphase3	0	1450.11	619.1622	1.65	0.77	0.61	1.13	0.52	2.28	0.71	32.5	21.59	45.91	46	12.4	49.7	47.6	15.1	0
CKphase4	0	1407.28	605.935	1.6	0.76	0.61	1.13	0.53	2.29	0.72	32.5	22	45.5	45.5	13.3	49.2	48.3	16	0
CKphase5	0	1429.144	585.31	1.6485	0.7865	0.6225	1.14077	0.5238	2.2836	0.71854	32.3	21.8	45.91642	45.5	12.5	49.3	47.2	15.4	0
LOphase1	0.63	1421.67	604.5657	1.61	0.78	0.62	1.13	0.52	2.25	0.71	32.28	22.35	45.37	45	12.1	49.9	46.6	15.1	1.5
LOphase2	1.33	1399.14	588.2281	1.62	0.78	0.62	1.14	0.53	2.26	0.71	32.15	22.09	45.76	45.1	12	49.9	46.7	14.9	1.7
LOphase3	2.03	1501.12	638.3518	1.63	0.79	0.62	1.13	0.53	2.26	0.71	31.23	22.11	46.66	46.5	12.3	49.7	48.1	14.8	0.1
LOphase4	2.53	1432.33	585.9087	1.67	0.82	0.63	1.13	0.53	2.23	0.72	30.23	22.51	47.26	44.6	11.2	50.4	46	14.1	2
LOphase5	3.03	1454.46	645.6587	1.69	0.77	0.64	1.15	0.54	2.28	0.72	30.53	22.05	47.42	44.5	12.2	49.2	46.1	15.3	2.2
MOphase1	1.4	1382.73	563.3891	1.7	0.81	0.63	1.14	0.55	2.25	0.72	28.95	22.77	48.28	45.4	13.7	49.2	47.5	16.8	1.1
MOphase2	2.52	1342.44	533.87	1.739	0.824	0.6595	1.15283	0.55603	2.2439	0.71823	27.84	22.9	48.2365	44.9	13.5	50	46.8	16.7	1.4
MOphase3	3.58	1495.33	635.8896	1.68	0.81	0.65	1.15	0.54	2.28	0.72	31.15	22.06	46.79	46.1	12	49.5	47.7	14.6	0.5
MOphase4	4.64	1401.47	629.2568	1.72	0.8	0.69	1.15	0.54	2.29	0.71	29.15	21.03	49.82	45.2	15.5	49.2	47.8	18.9	3.3
MOphase5	6.33	1422.65	622.5397	1.75	0.813	0.71	1.17	0.56	2.32	0.72	28.7	21.98	49.32	45.6	15.5	48.4	48.2	18.7	2.5
HOphase1	3.41	1429.752	604.84	1.763	0.8255	0.7695	1.1927	0.59637	2.3908	0.7239	28.04	21.96	50.00144	45.6	15.4	48.3	48.1	18.7	3.1
HOphase2	7.29	1399.22	618.7409	1.73	0.83	0.69	1.19	0.55	2.36	0.7	29.57	22.15	48.28	44.9	13.5	49.8	46.9	16.8	2.4
HOphase3	10.47	1385.81	596.2847	1.75	0.85	0.79	1.22	0.63	2.39	0.73	27.12	21.09	51.79	45.7	16	45.3	49.1	19.5	3.6
HOphase4	14.12	1400.55	580.7556	1.8	0.919	0.93	1.29	0.69	2.51	0.71	23.69	22	54.31	46.6	17.3	44.8	49.7	20.4	6
HOphase5	17.35	1353.856	558.07	1.8415	0.983	1.106	1.35017	0.78037	2.6753	0.69868	20.03	22.17	57.79781	46.4	17.2	44.8	49.5	20.4	6.5

Abbreviations: CK, anaerobic wine (0 mg O2/L per time); LO, low-level oxygenation wine (0.5-0.9 mg O2/L per time); MO, medium-level oxygenation wine (1-1.9 mg O2/L per time); HO, high-level oxygenation wine (3-3.9 mg O2/L per time); TCO, total consumed oxygen; TP, total phenol (Folin–Ciocalteu assays), which were expressed as gallic acid equivalents; MP,

monomeric pigments; SPP, small polymeric pigments; LPP, large polymeric pigments; WC, total color of free anthocyanins and anthocyanins eventually involved in bisulfite adducts; $CDRSO_2$, color due to derivatives resistant to SO_2 bleaching; CD, color density; polymeric, polymeric anthocyanins; monomeric, monomeric anthocyanins; copigmented, copigmented anthocyanins.

Table S5. Total oxygen consumption (mg/L) during treatment.

	CK	LO	MO	HO
initial	0	0	0	0
phase1	0	0.63	1.4	3.41
phase2	0	1.33	2.52	7.29
phase3	0	2.03	3.58	10.47
phase4	0	2.53	4.64	14.12
phase5	0	3.03	6.33	17.35

Abbreviations: initial, initial wine before oxidation treatment; CK, anaerobic wine (0 mg O2/L per time); LO, low-level oxygenation wine (0.5-0.9 mg O2/L per time); MO, medium-level oxygenation wine (1-1.9 mg O2/L per time); HO, high-level oxygenation wine (3-3.9 mg O2/L per time).
